# Help-Seeking, Support, and Engagement in Gestational Diabetes Mellitus Online Communities on Facebook: Content Analysis

**DOI:** 10.2196/49494

**Published:** 2024-02-26

**Authors:** Sheila Pham, Kate Churruca, Louise A Ellis, Jeffrey Braithwaite

**Affiliations:** 1 Australian Institute of Health Innovation Macquarie University North Ryde, Sydney Australia

**Keywords:** clinical management, communication, content analysis, engagement, Facebook, gestational diabetes, health communication, help-seeking behavior, mental distress, online communities, peer-support, self-disclosure, self-management, support

## Abstract

**Background:**

The prevalence of gestational diabetes mellitus (GDM) has drastically risen in recent years. For some, self-management includes the use of GDM online communities on Facebook. Such communities can fill gaps in information and support that participants are not able to access elsewhere to address unmet needs. Given the popularity of sharing information about pregnancy on Facebook and the documented benefits of diabetes online communities, the same may be true of GDM online communities.

**Objective:**

This study aimed to categorize and quantify what is being discussed in GDM Facebook groups, including informational and emotional help-seeking behavior, and how this support and engagement may be demonstrated by peers through comments and reactions.

**Methods:**

We sourced the data from the 2 largest Facebook groups focused on GDM in Australia. A summative content analysis was conducted on original posts across the 2 groups and coded for topics as well as help-seeking types. The coding scheme was based on the previous work of Liang and Scammon. Visible indicators of engagement, including the number of comments and “reactions,” were tabled and manually evaluated.

**Results:**

There were 388 original posts, and the analysis produced 6 topics: GDM self-management (199/388, 51.3%), GDM clinical management (120/388, 30.9%), preparing for birth (40/388, 10.3%), mental distress (35/388, 9%), birth announcement (29/388, 7.5%), and GDM journey reflections (21/388, 5.4%). Secondary coding of help-seeking type revealed more than half of the posts were informational help-seeking (224/388, 57.7%), while a small proportion were both informational and emotional help-seeking (44/388, 11.3%), and some (12/388, 3.1%) were emotional help-seeking only. Self-disclosure was identified as a fourth category, comprising almost a quarter of all posts (90/388, 23.2%). A total of 6022 comments were posted in response to the original posts, and there were 4452 reactions across all posts. Emotional help-seeking attracted the most comments per thread (mean 21.5, SD 19.8), followed by informational and emotional help-seeking (mean 20.2, SD 14.7), informational help-seeking (mean 15.6, SD 14.6), and self-disclosure (mean 14.3, SD 21.8). Across all help-seeking categories, few reactions occurred compared to comments; in contrast, self-disclosure attracted a large number of reactions (mean 9.4, SD 45.3).

**Conclusions:**

This is one of the first studies to examine peer support in a GDM online community on Facebook. Our findings suggest that active participants’ needs around information and support in relation to GDM are being somewhat met by peer-led online communities. Given the practical limitations of formal health care, including the provision of ongoing social support, it is important to recognize how GDM online communities can complement formal health care and help address unmet needs.

## Introduction

Accessing health information on the web nowadays includes social media such as Facebook and, increasingly, its “group” function. Globally, the number of people engaging with Facebook groups equates to around 1.8 billion people per month [1]. The group function of Facebook is described as “a place to connect, learn, and share with people who have similar interests” [2]. Among the many “similar interests” people have are health concerns as well as life experiences such as pregnancy.

Research on pregnancy and the internet suggests that Facebook is used by some for supportive and informational purposes [3]. Given this, it is not surprising that many pregnancy Facebook groups now exist, as well as those focused on complications of pregnancy such as gestational diabetes mellitus (GDM). GDM is defined as any degree of hyperglycemia recognized for the first time during pregnancy [4]. As a condition, it affects a significant and growing proportion of pregnant women around the world each year [5]. Although GDM prevalence has drastically risen, there has been limited examination of the attendant growth of GDM online communities, including Facebook groups [6].

People may join online health communities because their family and community support networks do not include relatable others undergoing similar experiences [7], and thus they do not receive the benefit of “peer-to-peer health care” [8]. Research on diabetes online communities has found they fill gaps in information and support that participants are not able to access elsewhere [9]. Online health communities can provide both informational and emotional support, which helps people actively cope with health-related problems [10]. A study about breast cancer for women suggested that patients specifically seek out discussion groups on the web due to “unmet needs” [11], while another study suggested online health communities are where a range of desires and needs can be met by peers [12]. A scoping review of 47 studies focused on the use of diabetes online communities found a variety of psychosocial benefits, and although reports of negative consequences were low, it was also noted that diabetes online communities may not be beneficial for all [13].

In online health communities, users often demonstrate support-seeking behavior through explicitly stated requests, with posting itself a signal that the person is a potential support provider to others [10]. It is also common for users to “share” or self-disclose as a coping mechanism [14]. On Facebook, in addition to initiating posts, active engagement occurs through comments and reactions such as “likes.” The meaning of these reactions is not necessarily explicit beyond face value but can be broadly interpreted as support [15], though arguably a comment is a stronger indicator of support given the greater time and effort required to produce it.

Given the popularity of sharing information about pregnancy on Facebook and the documented benefits of diabetes online communities, the same may also be true of GDM online communities. Furthermore, health information-seeking online can also improve the patient-physician relationship if the patient discusses the information with the physician and they have a positive prior relationship [16]. This may be worthwhile considering the context of GDM, which generally requires additional health care compared to pregnancies without complications.

The aims of this study about Facebook posts and interactions within GDM Facebook groups were to examine: (1) the issues being discussed, (2) evidence for informational and emotional help-seeking behavior, and (3) how this support and engagement is demonstrated through comments and reactions.

## Methods

### Study Design and Data Collection

This study sourced data from 2 peer-led closed Facebook groups focused on GDM, founded, and run independently by private individuals. All original posts (ie, the first post in a thread) during a 1-week period were included, as well as replies published during the collection week. A limited period was chosen because the large volume of posts was considered sufficiently robust for the purposes of this study. These particular Facebook groups were chosen as they were the 2 largest groups focused on GDM in Australia; at the time of data collection, the combined membership of the 2 groups was over 6500 members. For this study, a “snapshot” approach was taken, with the data set copied verbatim by the first author, then fully deidentified and recollated for analysis to protect the privacy of participants. The data were then analyzed using content analysis and descriptive statistics.

### Analysis

First, summative content analysis [17] was used to identify key topic areas from all “original posts” in a thread (ie, the first posts). This inductive approach to analyzing qualitative data started with reading through the data and identifying and quantifying certain words and content to understand contextual use before applying latent content analysis. To this end, the first author independently read and reread each post and identified keywords (eg, blood sugar and insulin) as the basis of topics. Multiple topics were allowed within a single post (ie, categories were not mutually exclusive). All authors then compared and confirmed the identified categories and interpretations.

Second, the original post in every thread was coded in terms of help-seeking type (Table 1). Here, a deductive coding scheme was used, following Liang and Scammon [10]. Visible indicators of engagement, including the number of comments, “likes,” and “reactions,” were tabled and manually evaluated by the first author (SP). A total of 2 secondary authors (KC and LAE) verified a sample (n=10) of the first author’s coding to ensure consistency. The depth of analysis was further consolidated by the research team, which compared and discussed codes to provide additional perspectives.

**Table 1:** Help-seeking coding scheme for original posts.

Categories	Description	Example^a^
Informational help-seeking	Asking for information (eg, suggestions or comments)	“Does anyone have any recommendations for a brand of bread that won’t spike my blood sugars?”
Emotional help-seeking	Expressing negative emotions (eg, embarrassment) to seek help	“I’m so stressed right now and need advice.”
Informational and emotional help-seeking	Asking for information and expressing negative emotions	“This feels so hard, not sure I can get through the next six weeks without losing it. What’s been helping you cope with this?”

^a^Exemplary but not direct quotes.

Finally, as the data from the 2 Facebook groups were combined to enlarge the sample size and potentially increase heterogeneity, it was important to determine whether there were statistically significant differences in proportions between the help-seeking categories of the 2 groups. A Fisher exact test was deemed an appropriate test given the likelihood of small category sample sizes.

### Ethical Considerations

Research based on Facebook posts raises important ethical questions, given the implications for privacy. Before the commencement of data collection, approval was sought and gained from Macquarie University’s Human Research Ethics Committee (5201827734364). When the first author (SP) requested permission from the administrators of both groups to join in order to conduct research, she disclosed her positionality as someone who had experienced GDM. As stipulated by the terms of the ethics approval, no identifying data would be published, including verbatim quotes.

## Results

### Topic Areas

A total of 388 original posts were extracted across the 2 groups, with 63 posts from one group and 325 posts from the other. From the content analysis, 6 topic areas were identified (Table 2). These were not mutually exclusive, as some longer posts were coded for more than 1 topic.

**Table 2:** Topics and descriptions.

Category	Description	Nonverbatim exemplars	Frequency, n (%)
GDM^a^ self-management	Questions and discussion relating to the day-to-day management of GDM, including blood sugar levels, diet, and equipment.	“Any advice on what to eat for supper to reduce your fasting levels?”	199 (51.3)
GDM clinical management	Questions and discussions relating to any aspect of formal GDM health care, including testing, diagnosis, scans, treatment, and patient-provider interactions.	“Had my 28-week scan today. Has anyone else had a baby measuring on the 95th percentile?”	120 (30.9)
Preparing for birth	Questions and discussion relating to birth including being induced (or fear thereof). Also discussion about baby’s sugar levels and expressing colostrum antenatally.	“When did everyone get an induction date?”	40 (10.3)
Mental distress	Overtly expressed distress relating to GDM.	“Feeling disheartened right now.”	35 (9)
Birth announcement	Announcing birth, typically with name, birth weight and other details. Often includes a photo and encouraging words.	“Introducing my sugar baby...”	29 (7.5)
GDM journey reflections	Sharing of overall GDM journey, including gratitude for the group and unexpected benefits.	“I wanted to share my GD journey with you all. Diagnosed at 28 weeks...”	21 (5.4)

^a^GDM: gestational diabetes mellitus.

A number of residual topics were excluded from the main analysis given the relatively small number of posts: “other pregnancy experiences” (n=18), “humor and memes” (n=9), “postpartum concerns” (n=7), “food and diet” (n=11), and “group management” (n=3).

### Help-Seeking and Engagement

Secondary coding of help-seeking type is captured in Table 3. The process identified mutually exclusive categories, where more than half of the posts were classifiable as informational help-seeking (224/388, 57.7%). A small proportion were classifiable as both informational and emotional help-seeking (44/388, 11.3%), while a minority (12/388, 3.1%) were emotional help-seeking only.

**Table 3:** Categories of original posts.

Categories	Frequency, n (%)	Comments, n	Comments per post, mean (SD)	Reactions, n	Reactions per post, mean (SD)
Informational help-seeking	224 (57.7)	3528	15.6 (14.6)	537	2.4 (20.1)
Informational and emotional help-seeking	44 (11.3)	887	20.2 (14.7)	60	1.4 (3.9)
Emotional help-seeking	12 (3.1)	258	21.5 (19.8)	32	2.7 (4.4)
Self-disclosure	90 (23.2)	1284	14.3 (21.8)	3550	39.4 (45.3)

Through the process of secondary coding we identified a distinct fourth category: self-disclosure. The intention behind such posts was not overt in terms of help-seeking (eg, “Just wanted to tell you all I gave birth to a healthy baby boy last week”). Almost a quarter of posts (90/388, 23.3%) were classifiable as self-disclosure.

A small number of posts (n=18) did not fit into any of the above 4 categories, such as posts sharing a recipe without comment or other practical matters such as offering to pass on medical supplies.

Visible indicators of engagement, namely the number of comments and reactions (including “likes”), were also tabled across all threads. A total of 6022 comments were posted in response to the original posts. The length of threads ranged from 1 to 179, with the median number of comments being 11. There were 4452 reactions across all posts. Emotional help-seeking posts were less prevalent but attracted the most comments per thread (mean 21.5, SD 19.8), followed by informational and emotional help-seeking (mean 20.2, SD 14.7), informational help-seeking (mean 15.6, SD 14.6), and self-disclosure (mean 14.3, SD 21.8).

Overall, across the 3 help-seeking categories, relatively few reactions occurred compared to comments, regardless of whether it was informational (mean 2.4, SD 20.1), informational and emotional help-seeking (mean 1.4, SD 3.9), or emotional help-seeking (mean 2.7, SD 4.4). In comparison, self-disclosure attracted a very large number of reactions (mean 39.4, SD 45.3).

### Significant Differences Between the Groups

A Fisher exact test was applied to determine if there were any statistically significant differences in the proportions of help-seeking categories between the 2 Facebook groups. There were no significant differences found between the groups except for “emotional help-seeking,” with 6 posts identified for both groups, which represented a statistically significant (*P*=.006) difference in the proportions of 9.5% (group 1) and 1.8% (group 2).

Upon closer examination of how emotional help-seeking posts were responded to in each group, there were other notable differences that further qualified this significant difference. In the smaller group (group 1), emotional help-seeking posts attracted far more comments in response (mean 26.8, SD 27) compared to the larger group (group 2), which had fewer comments in response to emotional help-seeking posts (mean 16.2, SD 7.9). Conversely, there were fewer reactions in group 1 (mean 1.8, SD 2.6) compared to group 2 (mean 3.5, SD 5.8).

## Discussion

### Overview

GDM self-management was the prevalent topic in over half the posts (199/388, 51.3%), which likely reflects a key motivation for both joining a GDM online community as well as an important reason for sustaining membership and engaging. The second most prevalent topic, GDM clinical management (120/388, 30.9%), is suggestive of the inadequacy of care provided in formal health care settings, including information provision, hence the need for additional discussion on the web with peers. This accords with how online health communities have been described as “communities of practice” due to the way learning occurs through a combination of experiential knowledge and other expert sources [18]. The topic “preparing for birth” alludes to a desire for information from peers (and expert sources), whereas “GDM journey reflections” points to expressly stated individual learning coupled with a desire to share and pass on knowledge to peers.

Informational was by far the most popular type of help-seeking, and this categorization largely overlapped with the most popular topics, demonstrating how critical information is in a peer-support context outside of formal health care. There were fewer posts where emotional help-seeking was the sole focus or in combination with informational help-seeking, but it is not surprising that these attracted the most comments per thread as empathic peers made a concerted effort to engage and offer reassuring words and engagement.

The statistically significant difference between the 2 Facebook groups in terms of the proportion of emotional help seekers warrants discussion. A possible explanation is the difference in size of the Facebook groups. Smaller-sized groups, in general, are friendlier and promote more contributions from members, with greater opportunities to speak [19], and emotional help-seeking posting also encourages supportive peers to show reciprocity by being more supportive. Examining the data from the 2 groups, there is a clear difference in terms of the volume of comments. This suggests greater intimacy and engagement in the smaller group, with comments being a better indicator of support than reactions, which are more impersonal and require less time and effort.

When we look at the posts categorized under “self-disclosure,” there are fewer comments but a much larger number of reactions. In such cases, engaging seems to be primarily enacted through a “reaction,” as peers do not necessarily see a need to comment. The general popularity of self-disclosure in GDM online communities is evident from this data and supports a more general observation that self-disclosing in an unprompted manner is intrinsic to the parlance of social media; that this is true of GDM online communities as many others. It has been found that self-disclosing in itself can be valuable, and with supportive conversation partners, there are positive psychological benefits, including reducing stress and improving self-affirmation [20]. In addition, previous research has found that even when someone only reads a poster’s self-disclosure without interaction, they can still develop a sense of personal connection [14]. It is useful to consider that self-disclosure can be further classified as per Malloch and Zhang [14], with factual self-disclosure revealing factual information, cognitive disclosure revealing thoughts and reasoning, and emotional disclosure revealing the poster’s feelings. This deeper categorization of self-disclosure was not undertaken in this study as it was beyond its scope, but merits further exploration in future research.

A final but nonetheless important consideration is the role of “lurkers,” who comprise the majority of participants in online communities; it is difficult to measure the true impact of interactions on all users of the groups because lurkers are not obvious [14]. For the most part, the visible support and engagement through comments and reactions is what users of a group are able to see. Given the large number of lurkers, however, we can only assume that the true engagement and utility of posts and comments are greater than what has been captured here. Furthermore, comment threads in groups can spark private messages between users, with discussions not visible to anyone else.

### Strengths and Limitations

There were a number of strengths in this study. Given the dearth of literature about GDM online communities, this research illuminates an emergent phenomenon and activity experienced by many thousands around the world. The findings suggest important avenues for further inquiry in relation to GDM, in both online and offline settings.

A key limitation is that the data were only analyzed based on visible comments and reactions. Another limitation is that emoticons and photos were not systematically coded, even though they were part of the data set, as the semiotics of both were beyond the scope of this analysis. Finally, only 1 week worth of data were analyzed, and the results may vary depending on the collection period.

### Conclusion

This study affirms the value of peer support that can be found in an online community. The large volume of posts and comments as well as high levels of positive engagement suggest that active participants’ needs around information and support in relation to GDM are being somewhat met by a peer-led online community. Given the practical limitations of formal health care, including the provision of ongoing social support, it is important to recognize how GDM online communities can complement health care and help address unmet needs. Furthermore, examining what information is being sought and shared by participants in GDM online communities is suggestive of gaps in information delivered through formal health care.

## Abbreviations

GDM: gestational diabetes mellitus.
